# 456. The Racial Divide: A Follow Up Study on Racial Disparity Amongst COVID-19 Survivors in an Urban Community in New Jersey

**DOI:** 10.1093/ofid/ofab466.655

**Published:** 2021-12-04

**Authors:** Christopher Millet, Humberto Jimenez, Emily Racoosin, George Horani, Yezin Shamoon, Spandana Narvaneni, Sherif Roman, Arslan Chaudhry, Sohail Chaudhry, Alisa Farokhian, Polina Aron, Christina Kmiecik, Beenish Faheem, Hamdallah Ashkar, Fady Shafeek, Patrick Michael, Jin Suh

**Affiliations:** 1 St Joseph’s University Medical Center, West Orange, New Jersey; 2 West Orange, New Jersey

## Abstract

**Background:**

We conducted a follow up study on patients previously diagnosed with COVID-19 one year ago in an urban community in Paterson, New Jersey. The purpose of the study was to evaluate the socioeconomic impact of COVID-19 as well as assess for receptiveness towards COVID-19 vaccination amongst various ethnic groups.

**Methods:**

This was a prospective cohort study consisting of patients who had COVID-19 in the months of March and April of 2020. This was a single institutional study conducted at St. Joseph’s Hospital in Paterson, NJ from March to April of 2021. Patients included were either male or female aged 18 years or older. Patients were contacted by telephone to participate to completed the survey. Chi-square testing and multivariable logistic regression analysis were utilized for statistical analysis.

**Results:**

Of the 170 patients enrolled in the study, the most common ethnicity was Hispanic (79/170 [46.47%]), followed by African American (46/170 [27.05%]). 83 patients were male (83/170 [48.82%]). Caucasians were the most willing to receive a COVID-19 vaccine (28/30 [93.3%]), followed by Asians (13/14 [92.8%]), Hispanics (63/78 [80.7%]) and African Americans (29/46 [63.0%]). Hispanics had the highest rate of job loss (31/79 [39.24%]), followed by African Americans (16/46 [34.7%]). Hispanics were found to be in the most financial distress (31/79 [39.2%]), followed by African Americans (17/46 [36.9%]). Hispanics and African Americans were more likely to refuse COVID-19 vaccination (p: 0.02). Hispanics were more likely to lose their jobs compared to Caucasians (odds ratio,4.456; 95% CI, 1.387 to 14.312; p: 0.0121). African Americans were also more likely to lose their jobs when compared to Caucasians (odds ratio, 4.465; 95% CI, 1.266 to 15.747; p: 0.0200).

Willingness amongst COVID-19 survivors to get vaccinated based on ethnicity

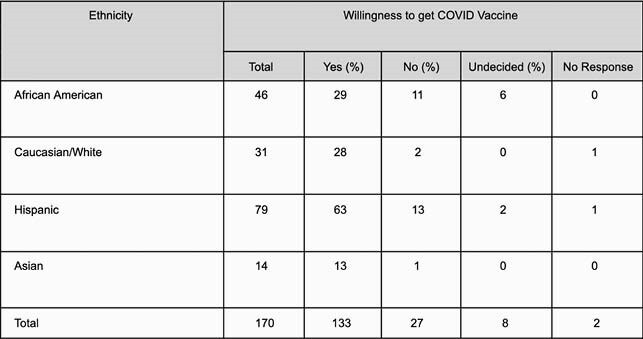

COVID-19 survivors who lost their jobs following diagnosis with COVID-19 based on ethnicity

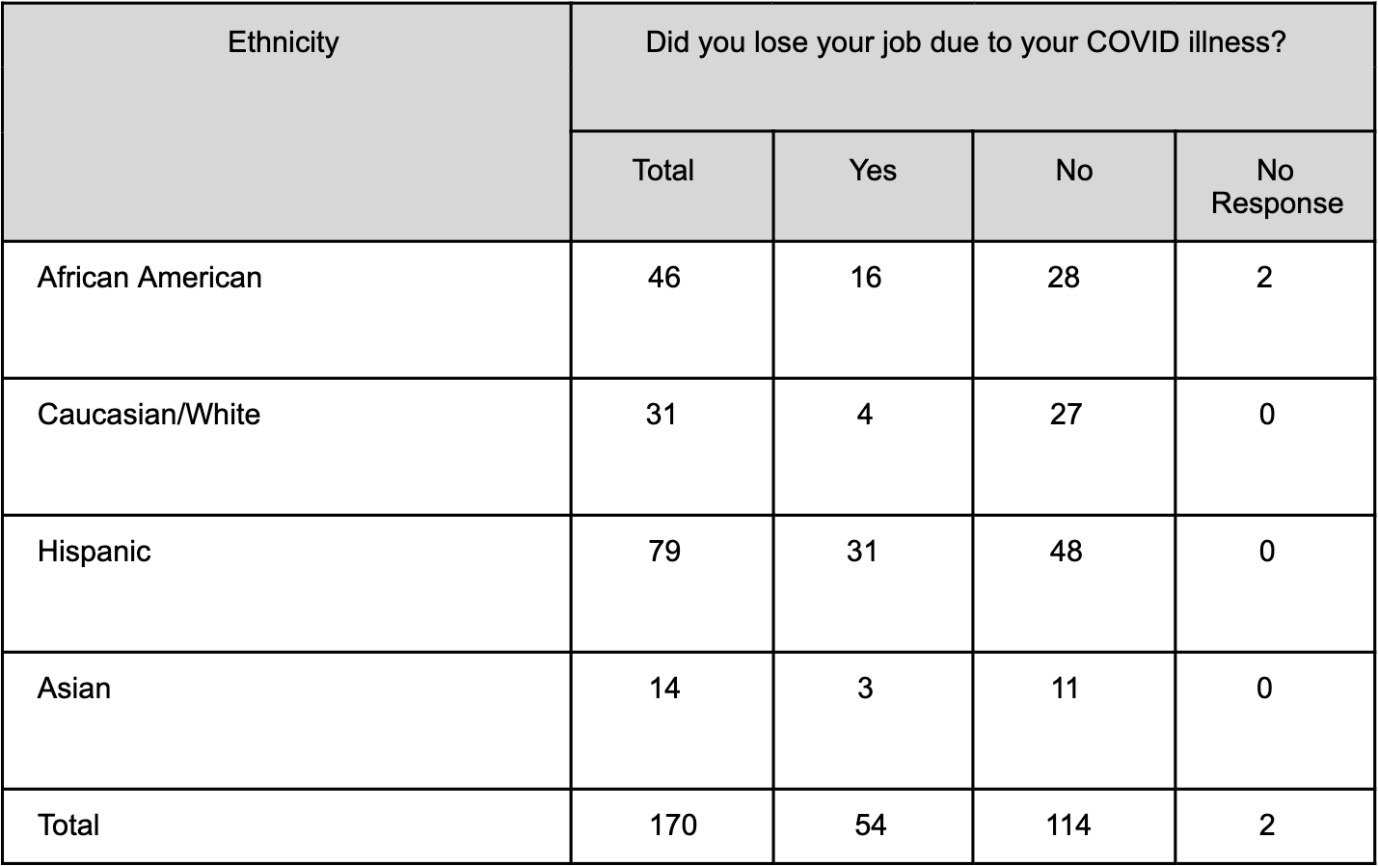

COVID-19 survivors who are experiencing financial distress following diagnosis with COVID-19 based on ethnicity

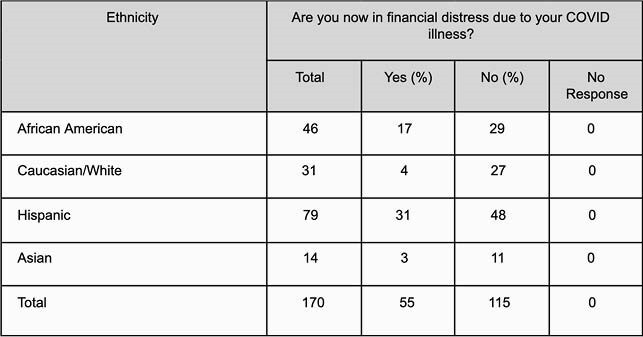

**Conclusion:**

Hispanics reported the most financial distress and with nearly 40% losing their jobs, the highest in our study group. 37% of African Americans experienced job loss and financial distress following their diagnosis with COVID-19. Only 63% of African Americans and 80.7% of Hispanics were willing to get vaccinated, mostly due to lack of trust in the vaccine. Statistical analysis showed Hispanics and African Americans were more likely to lose their jobs and refuse COVID-19 vaccination following diagnosis with COVID-19.

**Disclosures:**

**All Authors**: No reported disclosures

